# Bioactive Oxadiazoles 2.0

**DOI:** 10.3390/ijms23073841

**Published:** 2022-03-31

**Authors:** Antonio Palumbo Piccionello

**Affiliations:** Dipartimento di Scienze e Tecnologie Biologiche, Chimiche e Farmaceutiche-STEBICEF, Università degli Studi di Palermo, V.le delle Scienze Ed.17, 90128 Palermo, Italy; antonio.palumbopiccionello@unipa.it

Oxadiazoles are electron-poor, five-membered aromatic heterocycles that contain one oxygen and two nitrogen atoms. The oxadiazoles, namely 1,2,3-, 1,2,4-, 1,2,5-, and 1,3,4-regioisomers, together with N-oxides, benzo-fused, and non-aromatic derivatives, have a wide range of applications, from material science to explosives and bioactive compounds. In the latter field, there are many possibilities for their application, and oxadiazoles have been revealed to be active as antitumoral agents, neuroprotective compounds, antimicrobials, antivirals, antidiabetics, and so on. This Special Issue entitled “Bioactive Oxadiazoles 2.0” intended to offer a comprehensive view of the panorama of the potential applications of these compounds toward various diseases. This expectation was met, and many applications of different biologically active compounds were proposed by distinguished researchers.

The 1,3,4-oxadiazole motif, linked to a 2-sulfanylpyridine-3-carboxamide [1] or to pyrrolo[3,4-d]pyridazinone [2] was inserted into two new classes of hybrid compounds that exerted anti-inflammatory activity through selective inhibition versus cyclooxygenases (COX). Anti-inflammatory activity was also observed in indomethacin derivatives linked to the 1,3,4-oxadiazole-2-thiol scaffold with the ability to release nitric oxide [3]. The 1,3,4-isomer presented interesting bactericidal activity, which was reviewed by Glomb and Świątek [4] and was shown in newly designed 3-Acetyl-2,5-disubstituted-1,3,4-oxadiazolines active against *Staphylococcus* spp. [5].

Regarding the 1,2,4-isomer, some 1,2,4-oxadiazolyl-amides were discovered as antifungal and nematicidal compounds against *Sclerotinia sclerotiorum* and *Meloidogyne incognita* [6]. 1,2,4-Oxadiazoles were also employed as synthetic precursors of quinazolinones with anti-diabetic activity through a novel reductive rearrangement [7].

Additionally, benzofuroxans were presented in this issue due to their peculiar reactivity and applications. In fact, azidonitrobenzofuroxans were shown to react with 1,3-carbonyl compounds through Regitz diazo transfer [8], while benzofuroxans linked to an aminothiazole scaffold were evaluated for their anticancer activity [9]. 

As the Guest Editor of this Special Issue, I would like to acknowledge all of the authors for their generous participation and for the high scientific value of all of the manuscripts, as well as the editorial team at *IJMS* for their support during the management and production of all of the submissions.

## References

[1] Świątek P., Glomb T., Dobosz A., Gębarowski T., Wojtkowiak K., Jezierska A., Panek J.J., Świątek M., Strzelecka M. (2022). Biological Evaluation and Molecular Docking Studies of Novel 1,3,4-Oxadiazole Derivatives of 4,6-Dimethyl-2-sulfanylpyridine-3-carboxamide. Int. J. Mol. Sci..

[2] Peregrym K., Szczukowski Ł., Wiatrak B., Potyrak K., Czyżnikowska Ż., Świątek P. (2021). In Vitro and In Silico Evaluation of New 1,3,4-Oxadiazole Derivatives of Pyrrolo[3,4-d]pyridazinone as Promising Cyclooxygenase Inhibitors. Int. J. Mol. Sci..

[3] Sava A., Buron F., Routier S., Panainte A., Bibire N., Constantin S.M., Lupașcu F.G., Focșa A.V., Profire L. (2021). Design, Synthesis, In Silico and In Vitro Studies for New Nitric Oxide-Releasing Indomethacin Derivatives with 1,3,4-Oxadiazole-2-thiol Scaffold. Int. J. Mol. Sci..

[4] Glomb T., Świątek P. (2021). Antimicrobial Activity of 1,3,4-Oxadiazole Derivatives. Int. J. Mol. Sci..

[5] Paruch K., Biernasiuk A., Berecka-Rycerz A., Hordyjewska A., Popiołek Ł. (2021). Biological Activity, Lipophilicity and Cytotoxicity of Novel 3-Acetyl-2,5-disubstituted-1,3,4-oxadiazolines. Int. J. Mol. Sci..

[6] Liu D., Luo L., Wang Z., Ma X., Gan X. (2022). Design, Synthesis and Antifungal/Nematicidal Activity of Novel 1,2,4-Oxadiazole Derivatives Containing Amide Fragments. Int. J. Mol. Sci..

[7] Marzullo P., Vasto S., Buscemi S., Pace A., Nuzzo D., Palumbo Piccionello A. (2021). Ammonium Formate-Pd/C as a New Reducing System for 1,2,4-Oxadiazoles. Synthesis of Guanidine Derivatives and Reductive Rearrangement to Quinazolin-4-Ones with Potential Anti-Diabetic Activity. Int. J. Mol. Sci..

[8] Chugunova E., Gazizov A., Islamov D., Burilov A., Tulesinova A., Kharlamov S., Syakaev V., Babaev V., Akylbekov N., Appazov N. (2021). The Reactivity of Azidonitrobenzofuroxans towards 1,3-Dicarbonyl Compounds: Unexpected Formation of Amino Derivative via the Regitz Diazo Transfer and Tautomerism Study. Int. J. Mol. Sci..

[9] Chugunova E., Micheletti G., Telese D., Boga C., Islamov D., Usachev K., Burilov A., Tulesinova A., Voloshina A., Lyubina A. (2021). Novel Hybrid Compounds Containing Benzofuroxan and Aminothiazole Scaffolds: Synthesis and Evaluation of Their Anticancer Activity. Int. J. Mol. Sci..

